# Assessment of Bidirectional and Threshold-Response Associations Between Cognitive Function and Physical Performance: Nationwide Cross-Sectional Study

**DOI:** 10.2196/80575

**Published:** 2025-11-13

**Authors:** Huixiu Hu, Lanying Xie, Yuqing Hao, Yajie Zhao, Huanhuan Luo, Kang Yu, Chao Sun

**Affiliations:** 1 Department of Nursing, National Center of Gerontology, Institute of Geriatric Medicine, Chinese Academy of Medical Science, Beijing Hospital, 1 Da Hua Road, DongDan, Dongcheng District, Beijing, 100730, China, 86 85138512; 2 School of Nursing, Beijing University of Chinese Medicine, Beijing, China; 3 Beijing Hospital, National Center of Gerontology, Institute of Geriatric Medicine, Chinese Academy of Medical Sciences & Peking Union Medical College, Beijing, China; 4 Department of Cardiology, National Center of Gerontology, Institute of Geriatric Medicine, Chinese Academy of Medical Science, Beijing Hospital, Beijing, China; 5 School of nursing, Peking University, Beijing, China; 6 Department of Clinical Nutrition, Chinese Academy of Medical Science and Peking Union Medical College, Peking Union Medical College Hospital, Beijing, China

**Keywords:** cognitive function, physical performance, threshold effect, aging, bidirectional association

## Abstract

**Background:**

The global aging population faces increasing risks of cognitive and physical decline, yet the bidirectional and nonlinear dynamics between these domains remain poorly understood.

**Objective:**

This study aims to investigate the bidirectional and nonlinear associations between cognitive function and physical performance in older adults and to identify threshold effects and population subgroups at elevated risk.

**Methods:**

This multicenter study of 20,671 older adults in China analyzed the bidirectional asymmetric and threshold-response associations between cognitive and physical function using logistic or linear regression and restricted cubic spline, with subgroup and interaction analyses.

**Results:**

The study revealed an asymmetrical bidirectional relationship between cognitive function and physical performance, with cognitive impairment having a stronger association with physical function (Short Physical Performance Battery [SPPB], odds ratio [OR] 2.45, *P*<.05 vs 1.16,*P*=.051; gait speed: OR 2.36,*P*<.05 vs 1.89, *P*<.050). Restricted cubic spline analysis identified 3 key inflection points at Mini-Mental State Examination (MMSE) scores of 19, 24, and 29. When MMSE <24, cognitive improvement significantly protected physical performance (SPPB: OR 0.86, 95% CI 0.84‐0.87; gait speed: OR 0.84, 95% CI 0.82‐0.85). The critical intervention window was between MMSE 19‐24 (SPPB, OR 0.66, 95% CI 0.57-0.77). At MMSE ≥24, balance protection remained significant (OR 0.69, 95% CI 0.68‐0.70), but overall physical performance showed diminished effects, with SPPB demonstrating a weaker, marginal association (*P*=.051). A paradoxical reversal effect was observed for gait speed (OR 1.05, 95% CI 1.04‐1.07). High-income (OR 5.24, 95% CI 3.91‐7.02) and high-education subgroups (OR 3.98, 95% CI 1.48‐10.70) exhibited heightened vulnerability.

**Conclusions:**

This study demonstrates a bidirectional, asymmetric relationship between cognitive function and physical performance, identifies a nonlinear threshold effect, and highlights population heterogeneity.

## Introduction

The global population is aging at an accelerated rate, with the proportion of individuals aged ≥60 years projected to reach 2.1 billion by 2050 [1]. This demographic shift amplifies the dual burden of cognitive decline and physical disability, which synergistically exacerbate risks of falls, hospitalization, and dependency [2-4]. Cognitive function, encompassing memory, executive control, and information processing, deteriorates progressively from mild cognitive impairment to dementia. Concurrently, physical performance, as measured by the Short Physical Performance Battery (SPPB) [5], declines linearly with age, marked by reduced balance, gait speed, and muscle strength [6,7]. Critically, lower SPPB scores predict mortality and institutionalization [8-11], while cognitive impairment further compounds these risks through bidirectional pathways [4].

Extensive research [12-14] has demonstrated that impaired physical performance can significantly affect cognitive function, with slow gait speed being recognized as an early indicator for predicting cognitive decline and dementia. In addition, cognitive decline can also lead to impaired physical performance, with studies highlighting a bidirectional relationship between physical and cognitive health [15,16]. However, critical gaps persist. First, balance and muscle strength, key components of physical function, are rarely examined in relation to cognition. Second, evidence on bidirectionality is contradictory; one study [17] reported cognitive effects on gait speed but not balance, another linked balance deficits to cognitive impairment [18], and longitudinal data suggest cognitive decline drives physical deterioration more strongly than the reverse [15,19]. Third, prior studies predominantly assume linear relationships, neglecting potential thresholds where cognitive deterioration disproportionately escalates physical disability—a limitation obscuring precision intervention targets.

Our nationwide multicenter study explores bidirectional, nonlinear links between cognition and physical performance in older adults, identifying critical cognitive thresholds that increase physical risks. These findings guide precision interventions for dual decline, offering scalable strategies to reduce disability in aging populations.

## Methods

### Study Design

This study adopts a nationwide cross-sectional design to explore the relationship between cognitive function and physical performance, following the Strengthening the Reporting of Observational Studies in Epidemiology (STROBE) guidelines [20].

### Participants

A multistage stratified sampling method was used to recruit 20,868 community-dwelling adults aged 60+ years from 6 provinces in China (Fujian, Guangdong, Nanjing, Sichuan, Xinjiang, and Guizhou) between January and August 2022. The required sample size was calculated using the formula 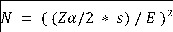 , yielding a sample size of 13,830 for a 95% CI with a margin of error of 0.05 and SD of 3 [21]. To account for invalid questionnaires, a 20% adjustment was made, resulting in a final sample size of 17,288. Inclusion criteria: participants aged 60+ with adequate communication and complete assessments. Exclusion criteria: participants with severe physical disabilities preventing SPPB testing or acute medical conditions (eg, postoperative recovery).

### Measures

#### Physical Performance

Physical performance was evaluated using the SPPB, a validated tool from the National Institute on Aging [22]. The SPPB comprises 3 subtests: balance, gait speed, and muscle strength, each component scored 0‐4, with a total score ranging from 0 to 12. Participants were classified as having normal (10–12) or poor (0‐9) physical performance [8,23]. In addition, a score of 4 in any subtest indicated normal function, while 0‐3 suggested impairment [24].

##### Balance Test

Three standing positions were feet-together, semi-tandem, and tandem. Point distribution was as follows: participants held the position for >10 seconds in feet-together or semi-tandem, earning them 1 point; standing for 3‐9.9 seconds in the tandem position earned 1 point, while standing for ≥10 seconds earned 2 points.

##### Gait Speed Test

The 2.44-meter walking speed test assigned scores based on speed: <0.43 m/s (1 point), 0.44‐0.60 m/s (2 points), 0.61‐0.77 m/s (3 points), and ≥0.78 m/s (4 points).

##### Muscle Strength Test

The Five-Times-Sit-to-Stand Test (FTSST) was used to assess lower limb muscle strength, with scores based on the time taken: 16.70‐60 seconds (1 point), 13.70‐16.69 seconds (2 points), 11.20‐13.69 seconds (3 points), and ≤11.19 seconds (4 points).

### Cognitive Function

Cognitive function was evaluated using the Mini-Mental State Examination (MMSE) [25], with the Chinese version validated by Wang et al [26] showing high test-retest reliability (intraclass correlation coefficient=0.91). The MMSE assesses five cognitive domains: orientation (time and place), memory (immediate and delayed recall), attention and calculation, language, and visuospatial ability. Scores ranged from 0 to 30, with higher scores indicating better cognitive function. The criteria for normal cognitive function vary by educational level: (1) middle school or above: score >24 (normal), ≤24 (impairment); (2) primary school: score >20 (normal), ≤20 (impairment); (3) illiteracy: score >17 (normal), ≤17 (impairment).

### Covariates

Sociodemographic characteristics and health behaviors were considered covariates. Sociodemographic characteristics included age, sex (male and female); BMI (<18.5, 18.5‐23.9, ≥24 kg/m^2^), educational level (primary school or below, middle school, high school or vocational school, and college or above), marital status (married vs other, including divorced, widowed, or single), number of children (none, 1, 2, 3, or more), residential area (rural, urban), living arrangement (living alone, living with relatives, living with a spouse), geographic region (eastern or western), data source (home-based or community health service center), and monthly household income (<5000 or ≥5000).

Health behaviors included the presence of chronic diseases (defined as having at least one: hypertension, coronary heart disease, cerebrovascular disease, or diabetes), long-term medications use (none, 1‐2, or ≥3), alcohol consumption (never, formerly, or currently), smoking status (never, formerly, or currently), weekly social participation frequency (none, 1‐3, or ≥4 d), weekly exercise frequency (none, 1‐3, or ≥4 d), self-reported social support (insufficient, inadequate, or sufficient), and self-reported visual and hearing impairment (no or yes).

### Data Collection

A designated assessor was assigned to each research site to oversee data collection using the “Jingyice Elderly Function Assessment Platform,” a WeChat (Tencent) mini-program developed by the research team. All assessors, registered nurses from community health centers, nursing homes, or hospitals, received uniform online training on the platform and study procedures. Before assessment, participants were informed of the study’s aims and procedures, and written informed consent was obtained. Assessors conducted face-to-face interviews and functional tests, entering responses and test results (eg, cognitive assessments and physical performance measures) into the mini-program, which synchronized data to a secure web-based platform for real-time monitoring. To ensure data quality, the backend recorded the time and duration of each evaluation, and the research team reviewed data weekly, focusing on submissions completed between 10 PM and 8 AM or within 15 minutes. Questionnaires and test records with incomplete scale data, >15% missing demographic information, patterned responses, or logical inconsistencies were excluded.

### Statistical Analysis

Continuous variables are presented as mean SD, while categorical variables are summarized as frequencies (%) via Pearson chi-square tests. To assess bidirectional associations, cognitive function (MMSE score) was modeled as both continuously and categorically (impaired or normal), whereas physical performance (SPPB total score) was analyzed as a continuous variable in linear regression and dichotomized (poor or normal) in logistic regression. All models were adjusted for covariates listed in the “Measures” section.

Nonlinear relationships were assessed using restricted cubic splines (RCS), with the optimal model fit chosen based on the Akaike Information Criterion from models with 3 to 6 knots. A 2-stage segmented linear regression approach identified threshold effects using the likelihood ratio test, and the optimal threshold was determined via an iterative grid search.

Subgroup analyses by sociodemographic factors (eg, age, education, and income) were conducted, and interaction effects were assessed using product terms (eg, MMSE×income). All analyses were performed with R (version 4.2; R Core Team) and SPSS (version 25.0; IBM Corp). Statistical significance was set at a 2-tailed *P* value <.05.

### Ethical Considerations

This study was approved by the Ethics Committee of Beijing Hospital (approval number 2023BJYYEC-446‐02). Written informed consent was obtained from all participants prior to participation. All data were anonymized, participants received no compensation, and no identifiable images are included in the article or supplementary materials. The study was conducted in accordance with the ethical standards of the Declaration of Helsinki.

## Results

### Baseline Characteristics

**Figure 1.**
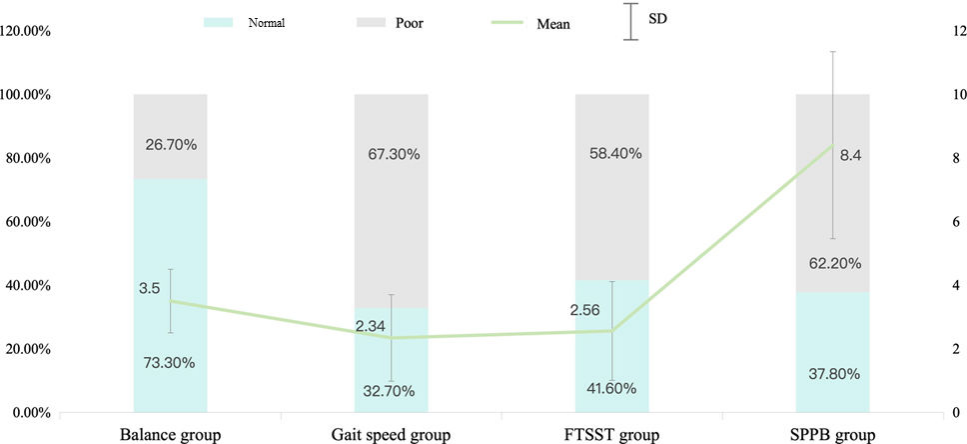
Physical function characteristics. FTSST: Five-Times-Sit-to-Stand Test; SPPB: Short Physical Performance Battery.

A total of 20,671 participants were included in the final analysis, with a mean age of 72.19 (SD 12.238) years; 48% (9931/20,671) were male and 52% (10,740/20,671) were female (Table 1). The majority of participants (12,703/20,671, 61.5%) had low education levels. Cognitive impairment was identified in 14.4% (2975/20,671), and 62.2% (12,857/20,671) exhibited poor physical performance with a mean SPPB score of 8.4 (SD 2.94). Domain-specific impairments were prevalent: 26.7% (5521/20,671) had balance deficits, 67.3% (13,906/20,671) showed reduced gait speed, and 58.4% (12,071/20,671) demonstrated muscle weakness, with gait speed showing the most pronounced deficit (mean score 2.34, SD 1.36; Figure 1). Furthermore, there were also differences in SPPB physical performance across various covariate subgroups (Table 1).

**Table 1.** Baseline characteristics.

Characteristics	Overall (N=20,671)	SPPB^a^		*P* value
		Normal (n=7804)	Poor (n=12,867)	
Age (years), mean (SD)	72.19 (12.238)	70.14 (11.791)	73.43 (12.337)	<.001
Sex, n (%)				.23
Male	9931 (48)	3791 (48.6)	6140 (47.7)	
Female	10,740 (52)	4013 (51.4)	6727 (52.3)	
BMI (kg/m^2^), n (%)				<.001
<18.5	1218 (5.9)	328 (4.2)	890 (6.9)	
18.5‐24	11,559 (55.9)	4326 (55.4)	7233 (56.2)	
≥24	7894 (38.2)	3150 (40.4)	4744 (36.9)	
Education, n (%)				<.001
Primary school or below	12,703 (61.5)	4229 (54.2)	8474 (65.9)	
Middle school	4401 (21.3)	1912 (24.5)	2489 (19.3)	
High school or vocational school	2635 (12.7)	1247 (16)	1388 (10.8)	
College or above	932 (4.5)	416 (5.3)	516 (4)	
Marital status, n (%)				<.001
Other	2271 (11)	708 (9.1)	1563 (12.1)	
Married	18,400 (89)	7096 (90.9)	11,304 (87.9)	
Children, n (%)				<.001
None	304 (1.5)	125 (1.6)	179 (1.4)	
One	4436 (21.5)	2123 (27.2)	2313 (18)	
Two	7164 (34.7)	2907 (37.3)	4257 (33.1)	
Three and more	8767 (42.4)	2649 (33.9)	6118 (47.5)	
Residential category, n (%)				<.001
Rural	9729 (47.1)	3369 (43.2)	6360 (49.4)	
Urban	10,942 (52.9)	4435 (56.8)	6507 (50.6)	
Living arrangement, n (%)				<.001
Living alone	1001 (4.8)	356 (4.6)	645 (5)	
Living with relatives	5833 (28.2)	2338 (30)	3495 (27.2)	
Living with spouse	13,837 (66.9)	5110 (65.5)	8727 (67.8)	
Region, n (%)				.62
Eastern	12,666 (61.3)	4765 (61.1)	7901 (61.4)	
Western	8005 (38.7)	3039 (38.9)	4966 (38.6)	
Source, n (%)				<.001
Home	11,920 (57.7)	4380 (56.1)	7540 (58.6)	
Community health service center	8751 (42.3)	3424 (43.9)	5327 (41.4)	
Income (yuan/month)^b^, n (%)				<.001
<5000	16,762 (81.1)	6032 (77.3)	10,730 (83.4)	
≥5000	3909 (18.9)	1772 (22.7)	2137 (16.6)	
Chronic diseases, n (%)				.79
None	7020 (34)	2659 (34.1)	4361 (33.9)	
Yes	13,651 (66)	5145 (65.9)	8506 (66.1)	
Types of long-term medications, n (%)				.14
None	8998 (43.5)	3453 (44.2)	5545 (43.1)	
1‐2	8321 (40.3)	3075 (39.4)	5246 (40.8)	
≥3	3352 (16.2)	1276 (16.4)	2076 (16.1)	
Alcohol, n (%)				<.001
Never	14,871 (71.9)	5415 (69.4)	9456 (73.5)	
Formerly	3761 (18.2)	1553 (19.9)	2208 (17.2)	
Currently	2039 (9.9)	836 (10.7)	1203 (9.3)	
Smoking, n (%)				<.001
Never	14,650 (70.9)	5419 (69.4)	9231 (71.7)	
Formerly	3195 (15.5)	1228 (15.7)	1967 (15.3)	
Currently	2826 (13.7)	1157 (14.8)	1669 (13)	
Social activity (d/wk), n (%)				<.001
None	5578 (27)	2021 (25.9)	3557 (27.6)	
1‐3	7538 (36.5)	2624 (33.6)	4914 (38.2)	
≥4	7555 (36.5)	3159 (40.5)	4396 (34.2)	
Exercise (d/wk), n (%)				<.001
None	5958 (28.8)	1966 (25.2)	3992 (31)	
1‐3	6443 (31.2)	2358 (30.2)	4085 (31.7)	
≥4	8270 (40)	3480 (44.6)	4790 (37.2)	
Social support, n (%)				.87
Insufficient	803 (3.9)	307 (3.9)	496 (3.9)	
Inadequate	1079 (5.2)	414 (5.3)	665 (5.2)	
Sufficient	18,789 (90.9)	7083 (90.8)	11,706 (91)	
Visual impairment, n (%)				.76
No	13,654 (66.1)	5165 (66.2)	8489 (66)	
Yes	7017 (33.9)	2639 (33.8)	4378 (34)	
Hearing impairment, n (%)				.27
No	17,696 (85.6)	6807 (87.2)	11,155 (86.7)	
Yes	2975 (14.4)	997 (12.8)	1712 (13.3)	
MMSE^c^, mean (SD)	25.51 (4.629)	26.54 (3.62)	24.88 (5.044)	<.001
Cognitive impairment, n (%)				<.001
No	17,696 (85.6)	7204 (92.3)	10,492 (81.5)	
Yes	2975 (14.4)	600 (7.7)	2375 (18.5)	

a SPPB: Short Physical Performance Battery.

b The exchange rate at the time of the study was US $1 = ¥7.113.

c MMSE: Mini-Mental State Examination.

### Bidirectional Relationship Between Cognitive Function and Physical Performance

#### Association of Cognitive Function on Physical Performance

Logistic regression models, adjusted for sociodemographic and behavioral covariates, demonstrated graded protective effects of cognitive function on physical performance. For each 1-point increase in the MMSE score, the risk of poor SPPB decreased by 7% (odds ratio [OR] 0.93, 95% CI 0.92‐0.93). Improvements were also observed in specific domains, including balance (OR 0.90, 95% CI 0.89‐0.90), gait speed (OR 0.94, 95% CI 0.94‐0.95), and muscle strength (OR 0.90, 95% CI 0.90‐0.91). Individuals with cognitive impairment had nearly double the risk of poor physical performance compared to those without (OR 2.45, 95% CI 2.22‐2.70), with gait speed (OR 2.36, 95% CI 2.13‐2.62) and muscle weakness (OR 2.23, 95% CI 2.03‐2.44) more strongly linked to cognitive impairment (Table 2).

**Table 2.** Logistic regression analysis of cognitive function on physical performance.

Models	SPPB^a^ group, OR (95% CI)	Balance group, OR (95% CI)	Gait speed group, OR (95% CI)	FTSST^b^ group, OR (95% CI)
Crude model^c^				
MMSE^d^	0.92(0.91-0.92)^e^	0.89 (0.88-0.89)^e^	0.94 (0.93-0.95)^e^	0.89 (0.89-0.90)^e^
Cognitive impairment				
No	Reference	Reference	Reference	Reference
Yes	2.72 (2.47-2.99)^e^	1.90 (1.75-2.06)^e^	2.50 (2.27-2.76)^e^	2.61 (2.38-2.85)^e^
Model 1^f^				
MMSE	0.93 (0.92-0.94)^e^	0.90 (0.89-0.90)^e^	0.95 (0.94-0.95)^e^	0.90 (0.90-0.91)^e^
Cognitive impairment				
No	Reference	Reference	Reference	Reference
Yes	2.43 (2.21-2.68)^e^	1.63 (1.49-1.78)^e^	2.36 (2.13-2.61)^e^	2.24 (2.04-2.46)^e^
Model 2^g^				
MMSE^d^	0.93 (0.92-0.93)^e^	0.90 (0.89-0.90)^e^	0.94 (0.94-0.95)^e^	0.90 (0.90-0.91)^e^
Cognitive impairment				
No	Reference	Reference	Reference	Reference
Yes	2.45 (2.22-2.70)^e^	1.62 (1.48-1.76)^e^	2.36 (2.13-2.62)^e^	2.23 (2.03-2.44)^e^

a SPPB: Short Physical Performance Battery.

b FTSST: Five-Times-Sit-to-Stand-Test.

c Crude model: unadjusted covariates.

d MMSE: Mini-Mental State Examination.

e *P*<.001.

f Model 1: adjusted for sociodemographic characteristics adjusted for sociodemographic characteristics (age, gender, BMI, education level, marital status, number of children, residential category, living arrangement, geographic region, data source, monthly household income).

g Model 2: adjusted for Model 1 variables plus health and behavioral factors(chronic diseases, types of long-term medication, alcohol consumption, smoking, frequency of weekly social activities, frequency of weekly exercise, self-reported social support, and self-reported vision and hearing impairments).

Linear regression analyses corroborated these findings. After full adjustment, each MMSE point increase predicted higher physical performance scores (SPPB: *β*=0.18, 95% CI 0.17‐0.19; balance: *β*=0.05, 95% CI 0.05‐0.06; gait speed: *β*=0.04, 95% CI 0.04‐0.05; FTSST: *β*=0.08, 95% CI 0.07‐0.08; all *Ps*<.001). Cognitive impairment persisted as a robust predictor of physical decline (SPPB: *β*=−1.35, 95% CI −1.46 to −1.24, *P*<.001) (Table S1 in Multimedia Appendix 1).

#### Association of Physical Performance on Cognitive Function

After adjusting for all covariates, the association of physical performance on cognitive function was further examined, revealing a significant impact on cognitive health (Table 3). Better physical performance, particularly better gait speed scores (OR 0.82, 95% CI 0.79‐0.85), was associated with a reduced risk of cognitive impairment. The SPPB impairments showed a marginal association with cognitive status (OR 1.16, 95% CI 1‐1.34; *P*=.051), while balance impairments (OR 1.71, 95% CI 1.57‐1.88), gait speed limitations (OR 1.89, 95% CI 1.66‐2.16), and muscle weakness (OR 1.66, 95% CI 1.47‐1.86) were significantly associated with cognitive impairment (*P*<.001).

**Table 3.** Logistic regression analysis of physical performance on cognitive function.

Models	Cognitive impairment, OR (95%CI)
Crude model^a^	
Balance	0.84 (0.81‐0.87)^b^
Gait speed	0.83 (0.80‐0.85)^b^
FTSST^c^	0.88 (0.86‐0.91)^b^
SPPB^d^	0.85 (0.84‐0.86)^b^
Balance group	1.84 (1.69‐2.00)^b^
Gait speed group	1.88 (1.66‐2.14)^b^
FTSST group	1.86 (1.66‐2.07)^b^
SPPB group	1.18 (1.03‐1.36)^e^
Model 1^f^	
Balance	0.88 (0.84‐0.91)^b^
Gait speed	0.82 (0.79‐0.90)^b^
FTSST	0.86 (0.831‐0.89)^b^
SPPB	0.85 (0.84‐0.86)^b^
Balance group	1.74 (1.59‐1.90)^b^
Gait speed group	1.89 (1.66‐2.15)^b^
FTSST group	1.69 (1.50‐1.90)^b^
SPPB group	1.14 (0.98‐1.31)
Model 2^g^	
Balance	0.88 (0.85‐0.92)^e^
Gait speed	0.82 (0.79‐0.85)^b^
FTSST	0.86 (0.83‐0.89)^b^
Balance group	1.71 (1.57‐1.88)^b^
Gait speed group	1.89 (1.66‐2.16)^b^
FTSST group	1.66 (1.47‐1.86)^b^
SPPB group	1.16 (1.00‐1.34)

a Crude model: unadjusted covariates.

b *P*<.001.

c FTSST: Five-Times-Sit-to-Stand-Test.

d SPPB: Short Physical Performance Battery.

e *P*<.05.

f Model 1: adjusted for sociodemographic characteristics adjusted for sociodemographic characteristics (age, gender, BMI, education level, marital status, number of children, residential category, living arrangement, geographic region, data source, monthly household income).

g Model 2: adjusted for Model 1 variables plus health and behavioral factors (chronic diseases, types of long-term medication, alcohol consumption, smoking, frequency of weekly social activities, frequency of weekly exercise, self-reported social support, and self-reported vision and hearing impairments).

Linear regression analysis confirmed our findings. In the fully adjusted model, higher scores in the SPPB, balance, gait speed, and muscle strength were associated with higher MMSE scores, while individuals with impaired physical performance had lower MMSE scores (*P*<.001), with balance ability showing the strongest association (*β*=1.01, 95% CI 0.96‐1.07). Those with balance impairments had an average MMSE score 2.02 points lower than those with normal balance (95% CI −2.15 to −1.89; Table S2 in Multimedia Appendix 1). These findings highlight gait speed and balance as key biomarkers for cognitive risk stratification.

This study shows a significant bidirectional and asymmetric relationship between cognitive function and physical performance. Cognitive impairment strongly affects physical performance, increasing the risk of SPPB (OR=2.45, 95% CI 2.22‐2.70), gait speed (OR=2.36, 95% CI 2.13‐2.62), muscle strength deficits (OR=2.32, 95% CI 2.03‐2.44), and balance impairments (OR=1.16, 95% CI 1‐1.34). In contrast, physical performance has a weaker association with cognitive function, with balance ability (OR 1.71, 95% CI 1.57‐1.88) being the strongest, and gait speed (OR 1.89, 95% CI 1.66‐2.16), muscle strength (OR 1.66, 95% CI 1.47‐1.86), and SPPB showing weaker, marginal associations (*P*=.051). This asymmetry suggests that maintaining cognitive health can better support physical function in older adults, offering dual benefits for both.

### RCS and Threshold Effect Analysis of Cognitive Function on Physical Performance

**Figure 2.**
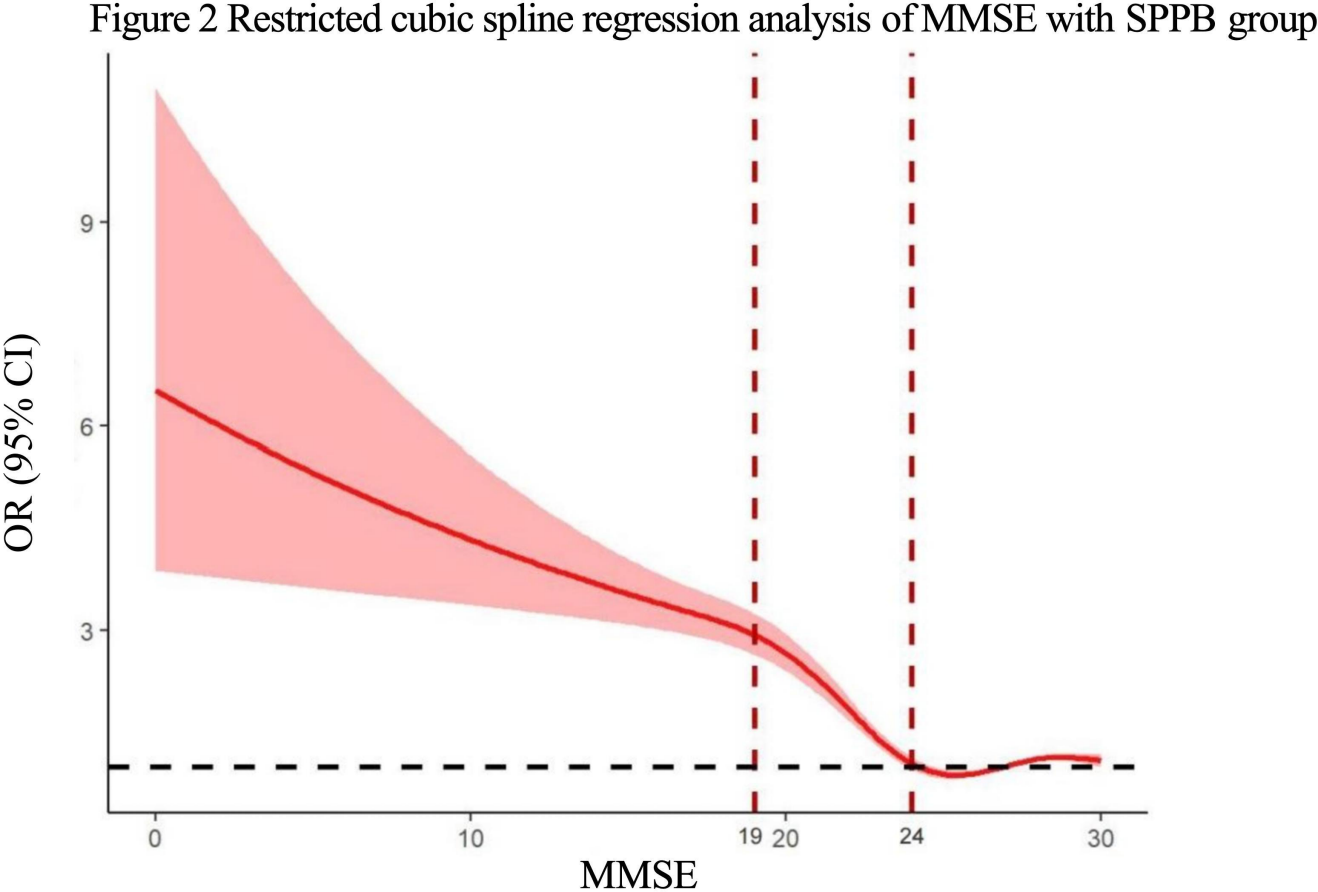
Restricted cubic spline regression analysis of Mini-Mental State Examination with Short Physical Performance Battery group. MMSE: Mini-Mental State Examination; SPPB: Short Physical Performance Battery.

**Figure 3.**
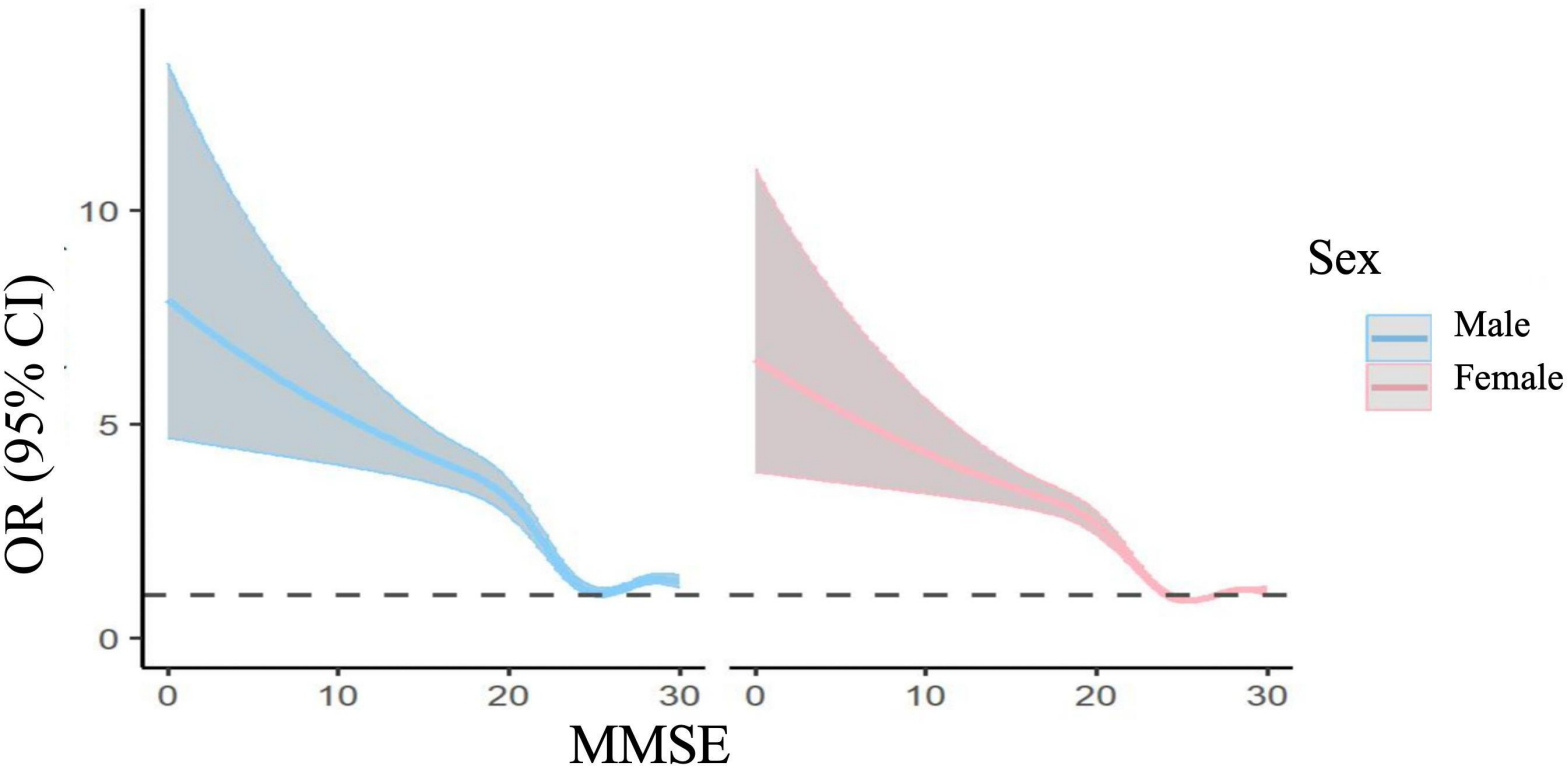
Gender stratification. MMSE: Mini-Mental State Examination.

RCS analyses showed significant nonlinear associations between cognitive function (MMSE) and physical performance measures (SPPB, gait speed, balance, and muscle strength; Figure 2 ; Figures S1-S3 in Multimedia Appendix 1). These relationships remained consistent in gender and education-stratified analyses (Figures3  4; Figures S1A-S3B in Multimedia Appendix 1), confirming robustness across demographic subgroups.

**Figure 4.**
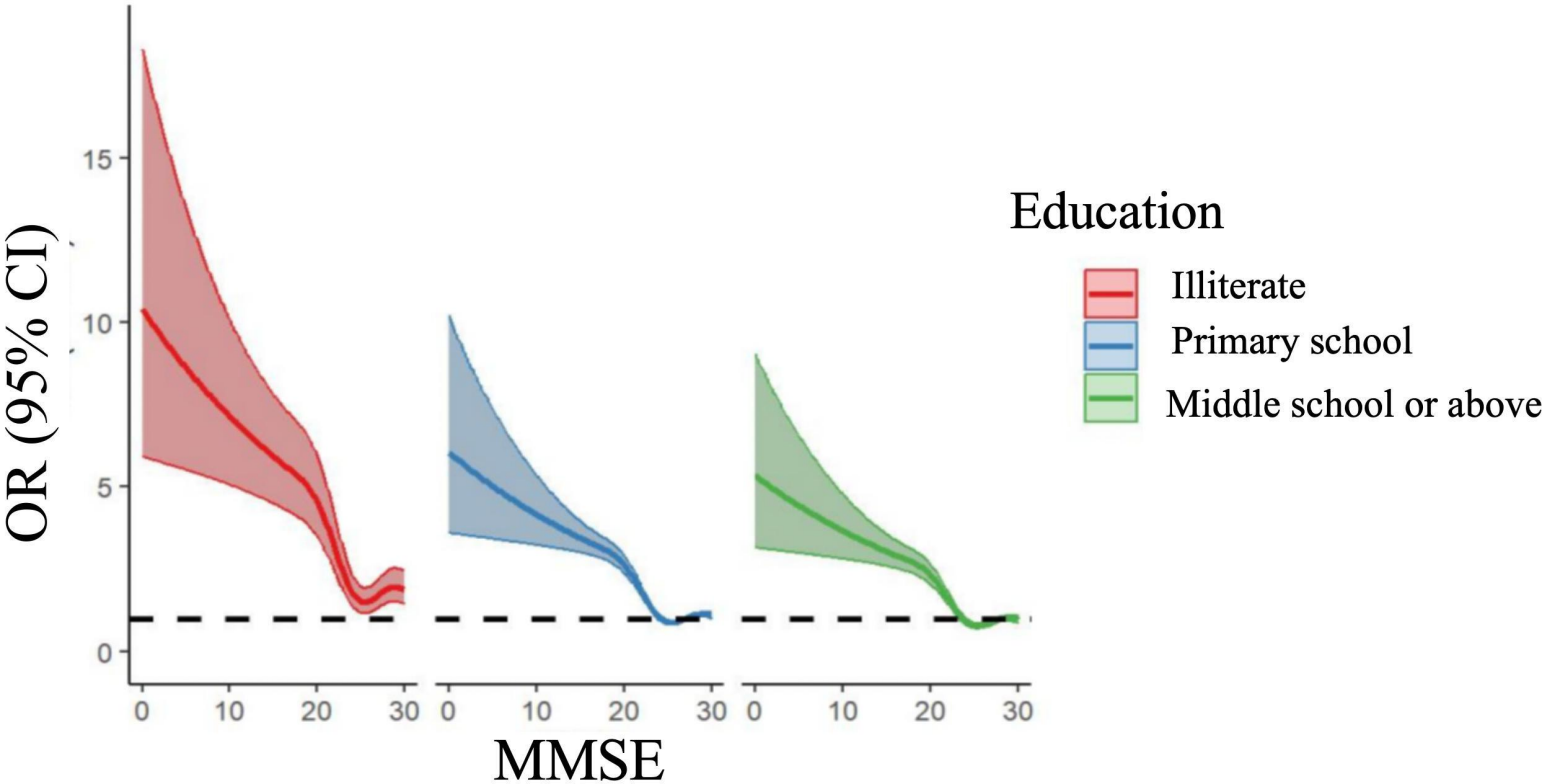
Education level stratification. MMSE: Mini-Mental State Examination.

This study revealed threshold effects and dynamic changes between cognitive function and physical performance using RCS analyses. Significant inflection points were found at MMSE=24 for overall performance (SPPB), balance, and gait speed, and at MMSE=29 for FTSST (Table 4). In the impaired cognitive period (MMSE <24), cognition improvements protected physical performance, reducing the risk of SPPB by 14% (OR 0.86, 95 CI 0.84‐0.87), gait speed by 16% (OR 0.84, 95% CI 0.82‐0.85), and balance by 4.8% (OR 1.05, 95% CI 1.03‐1.06). In the optimal cognitive period (MMSE ≥24), the protective association remained significant for balance (risk reduced by 31%, OR 0.69, 95% CI 0.68‐0.70) but weakened for overall performance (SPPB,*P*=.054), and a marginal positive association was observed for gait speed (OR 1.05, 95% CI 1.04-1.07), likely because cognitively intact individuals were already near the upper limit of normal performance, rather than reflecting a harmful relationship. The protective association on muscle strength persisted, with the most significant impact at MMSE >29 (risk reduced by 32%; OR 0.68, 95% CI 0.63‐0.74).

**Table 4.** Threshold effect analysis of cognitive function, MMSE^a^ on physical function.

Groups^b^	OR (95% CI)	*P* value
SPPB^c^ group		
Fitting by the standard linear model	0.92 (0.91‐0.93)	<.001
Fitting by the two-piecewise linear model	—^d^	—
Inflection point (score=24)		
MMSE^a^<24	0.86 (0.84-0.87)	<.001
MMSE≥24	1.00 (0.98-1.01)	.54
*P* for likelihood ratio	<.001	—
Balance group		
Fitting by the standard linear model	0.90 (0.89-0.90)	<.001
Fitting by the two-piecewise linear model	—	—
Inflection point (score=24)		
MMSE<24	1.05 (1.03-1.06)	<.001
MMSE≥24	0.69 (0.68-0.70)	<.001
*P* for likelihood ratio	<.001	—
Gait speed group		
Fitting by the standard linear model	0.94 (0.94-0.95)	<.001
Fitting by the two-piecewise linear model	—	—
Inflection point (score=24)		
MMSE<24	0.84 (0.82-0.85)	<.001
MMSE≥24	1.05 (1.04-1.07)	<.001
*P* for likelihood ratio	<.001	—
FTSST^e^ group		
Fitting by the standard linear model	0.90 (0.90-0.91)	<.001
Fitting by the two-piecewise linear model	—	—
Inflection point (score=29)		
MMSE<29	0.92 (0.91-0.93)	<.001
MMSE≥29	0.68 (0.63-0.74)	<.001
*P* for likelihood ratio	<.001	—

a MMSE: Mini-Mental State Examination.

b Adjusted for age, gender, body mass index (BMI), education level, marital status, number of children, residential category, living arrangement, geographic region, data source, monthly household income, chronic diseases, types of long-term medication, alcohol consumption, smoking, frequency of weekly social activities, frequency of weekly exercise, self-reported social support, and self-reported vision and hearing impairments.

c SPPB: Short Physical Performance Battery.

d Not applicable.

e FTSST: Five-Times-Sit-to-Stand-Test.

In addition, we identified a second inflection point in over physical performance (SPPB) at MMSE=19 using second derivative analysis (Figure S4 in Multimedia Appendix 1). After adjusting for confounding factors, the MMSE 19‐24 group showed a 34% lower risk of physical performance impairment (SPPB) compared to the MMSE <19 group (OR 0.66, 95% CI 0.57‐0.77) (Table S3 in Multimedia Appendix 1).

### Subgroup Analysis and Interaction Analysis

**Figure 5.**
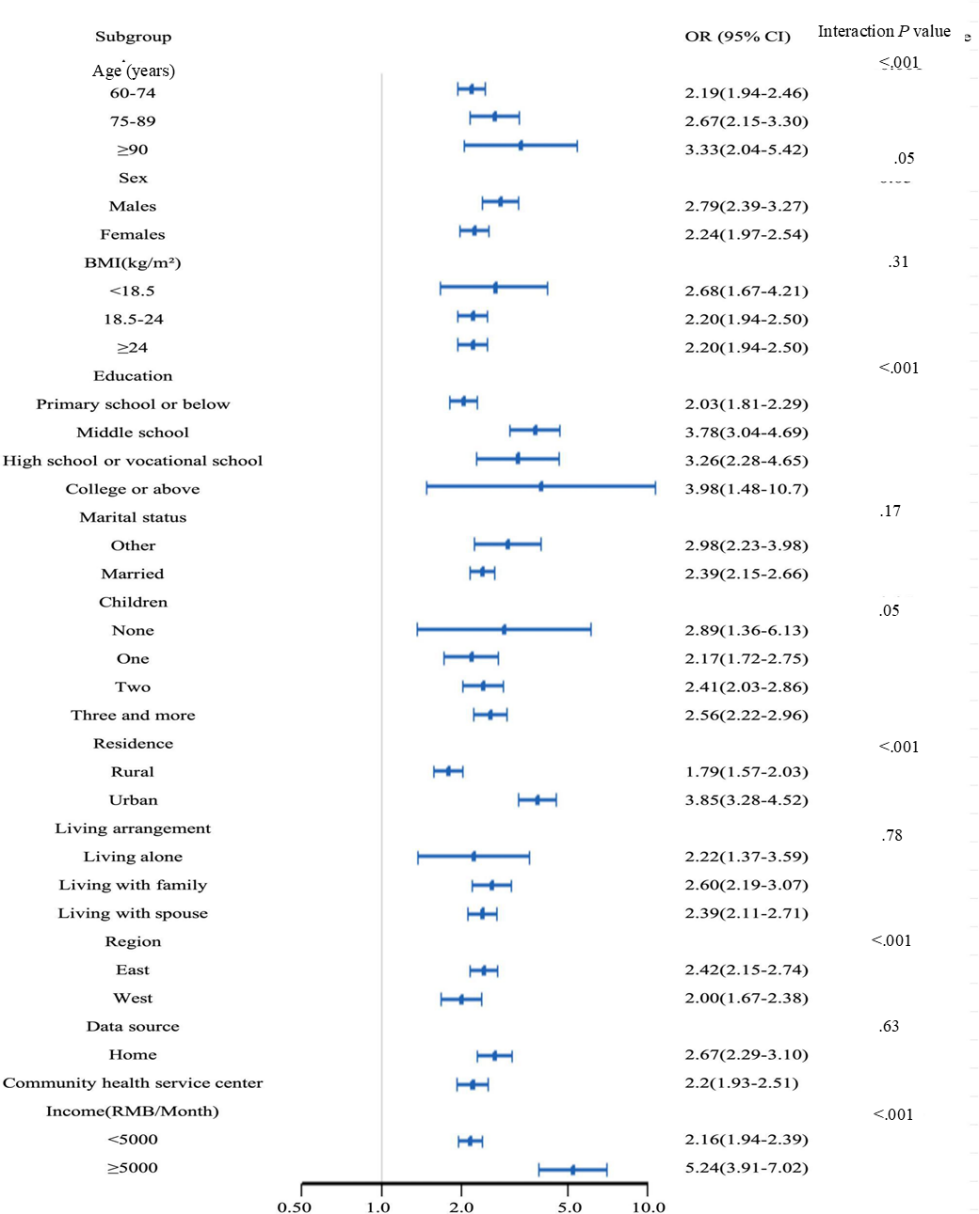
Subgroup and interaction analyses between cognitive function and physical performance across various subgroups.

Subgroup analyses revealed significant heterogeneity in the association between cognitive impairment and physical performance across demographic and socioeconomic strata (Figure 5). The strongest associations were observed in older adults aged ≥90 years (OR 3.33, 95% CI 2.04‐5.42), those with college or above level education (OR 3.98, 95% CI 1.48‐10.70), high-income households (≥5000 yuan per month [US $1 = ¥7.113]: OR 5.24, 95% CI 3.91‐7.02), and urban residents (OR 3.85, 95% CI 3.28‐4.52). Interaction models further demonstrated graded effects: the detrimental impact of cognitive impairment increased with advancing age (*β*=0.45‐0.71 per age group), higher education (*β*=0.55‐0.77), and socioeconomic advantage (*β*=0.64‐0.85 for urban residency and high income), suggesting these factors may amplify vulnerability to physical decline (Table S4 in Multimedia Appendix 1).

## Discussion

### Principal Findings

This study advances the understanding of the cognitive-physical performance relationship through 3 key innovations. First, we demonstrated a bidirectional yet asymmetric relationship: cognitive impairment exerted a stronger detrimental association on physical function than vice versa. Second, our nonlinear threshold analysis revealed critical inflection points (MMSE=19, 24, 29), defining distinct intervention phases. The MMSE 19‐24 range marked a critical intervention window where cognitive improvements maximally protected physical function, whereas MMSE >24 signaled a ceiling effect for global physical performance and a paradoxical reversal in gait speed, aligning with the cautious gait hypothesis. Finally, subgroup analyses uncovered a socioeconomic paradox: higher socioeconomic status (SES), indicated by advanced education, income, and living in an urban area, exhibited heightened vulnerability to physical functional decline. These innovations combine mechanistic research with clinical applications, offering strategies to address the disability burden in an aging society.

Our findings corroborate and extend the evidence on cognitive-physical interdependencies, while challenging the prevailing assumption of symmetrical reciprocity. Longitudinal studies have shown cognitive function as a predictor of physical decline, particularly fall risk [27] and gait deterioration [28]. A cross-sectional study [29] found that increased cognitive impairment severity leads to worsened balance control, consistent with our observation of bidirectional associations. Notably, while Li et al [28] reported a bidirectional relationship between cognitive function and gait speed, our analysis revealed a critical asymmetry: cognitive impairment exerted a disproportionately stronger impact on physical disability than the reverse. This divergence challenges the notion of equivalent bidirectional association and positions cognitive preservation as a pivotal strategy to disrupt the disability cascade.

This study found a nonlinear relationship between cognitive function and physical performance across individuals of various genders and educational levels. It identified 3 protective thresholds at scores of 19, 24, and 29, offering key evidence for developing precise intervention strategies to maintain functionality in older individuals. At the stage where cognitive function may be impaired (MMSE <24), it significantly protects all physical performance dimensions, with the greatest association observed in the 19‐24 score range, suggesting it should be a target for clinical intervention. These results align with previous research [30], which found a positive correlation between cognitive and physical function, suggesting cognitive intervention can delay physical decline. Our study further quantifies cognitive function threshold points. Thus, it is recommended to assess functional levels at this stage and conduct adaptive dual-task training targeting both cognitive and physical functions to enhance both [31].

In the optimal cognitive function stage (MMSE ≥24), physical performance exhibits dimension-specific changes: overall physical performance (SPPB) shows a clear “ceiling effect,” suggesting that further improvements in physical performance may have reached physiological limits, leaving limited room for cognitive function to contribute. Gait speed, however, exhibits an unusual reversal phenomenon, consistent with George et al [32] inverted U-shaped gait-cognition curve, attributed to the “cautious gait hypothesis.” This hypothesis suggests that older adults with higher cognitive function consciously regulate their movements, resulting in a more cautious and controlled walking pattern [33]. Cognitive function exerts a continuous protective association on balance ability, most pronounced when MMSE >29. This finding supports the view of Kuan et al [34] that early-stage mild cognitive impairment, regardless of musculoskeletal function, can impact balance, potentially due to the weakening of the cortical-vestibular network in individuals with cognitive impairment, particularly in key brain areas involved in integrating visual, auditory, and vestibular signals, correlating with the decline in balance ability [35].

Interestingly, our subgroup and interaction analyses revealed a socioeconomic paradox:

older individuals with higher SES, as measured by education and income, exhibited amplified risks of concurrent cognitive-physical decline, contradicting the conventional SES-health gradient. This counterintuitive association necessitates a multidimensional interpretation rooted in the Cognitive Reserve Hypothesis. While enhanced neurocompensatory mechanisms in high-SES populations may initially delay clinical manifestations of cognitive impairment [36], prolonged compensatory demands could deplete neural resources, precipitating accelerated physical deterioration once pathological thresholds are breached—a” double-edged sword” effect [37]. Moreover, behavior patterns related to occupational characteristics warrant attention. This group may have maintained an imbalanced “high cognitive load-low physical activity” state throughout their careers, leading to accelerated degeneration of motor function neural circuits [38]. These insights advocate for dual-target interventions prioritizing simultaneous cognitive reinforcement and physical activation in high-SES older adults, particularly during the identified critical window (MMSE 19‐24), to counteract reserve depletion trajectories.

Of course, our study has several limitations. First, as this study uses a cross-sectional design, future research should adopt longitudinal approaches to explore causal relationships and developmental trajectories. Second, due to the feasibility constraints of large-scale studies, many variables were assessed via self-report measures, potentially introducing reporting bias. Third, we did not directly measure occupational type or physical activity levels using validated instruments. Future studies are recommended to further investigate the “high cognitive load–low physical activity” hypothesis. Finally, this study evaluated overall cognitive function but did not assess specific cognitive domains, such as memory or executive function. Future research should investigate these domains to better understand the relationship between cognitive function and physical performance.

### Conclusions

This study demonstrates a bidirectional, asymmetric relationship between cognitive function and physical performance, identifies a nonlinear threshold effect, and highlights substantial population heterogeneity. These findings offer novel insights into the interaction between cognitive decline and physical disability, particularly in older individuals, and provide guidance for developing targeted interventions for high-risk subgroups in an aging society.

## Supplementary material

10.2196/80575 Multimedia Appendix 1 Multiple linear and restricted cubic spline regression analyses of cognitive function and physical performance, including inflection points, interaction effects, gender- and education-stratified analyses, and a second derivative analysis of the spline curve.

## Abbreviations

MMSE: Mini-Mental State Examination
OR: odds ratio
RCS: restricted cubic spline
SES: socioeconomic status
SPPB: Short Physical Performance Battery
