# Correction: Assessment of Bidirectional and Threshold-Response Associations Between Cognitive Function and Physical Performance: Nationwide Cross-Sectional Study

**DOI:** 10.2196/88582

**Published:** 2025-12-03

**Authors:** Huixiu Hu, Lanying Xie, Yuqing Hao, Yajie Zhao, Huanhuan Luo, Kang Yu, Chao Sun

**Affiliations:** 1 Department of Nursing, National Center of Gerontology, Institute of Geriatric Medicine, Chinese Academy of Medical Science, Beijing Hospital, 1 Da Hua Road, DongDan, Dongcheng District, Beijing, 100730, China, 86 85138512; 2 School of Nursing, Beijing University of Chinese Medicine, Beijing, China; 3 Beijing Hospital, National Center of Gerontology, Institute of Geriatric Medicine, Chinese Academy of Medical Sciences & Peking Union Medical College, Beijing, China; 4 Department of Cardiology, National Center of Gerontology, Institute of Geriatric Medicine, Chinese Academy of Medical Science, Beijing Hospital, Beijing, China; 5 School of nursing, Peking University, Beijing, China; 6 Department of Clinical Nutrition, Chinese Academy of Medical Science and Peking Union Medical College, Peking Union Medical College Hospital, Beijing, China

In “Assessment of Bidirectional and Threshold-Response Associations Between Cognitive Function and Physical Performance: Nationwide Cross-Sectional Study” [1], the authors noted one error in the abstract.

In the original paper, the following information was present:

This multicenter study of 20,868 older adults in China analyzed the bidirectional asymmetric and threshold-response associations between cognitive and physical function using logistic or linear regression and restricted cubic spline, with subgroup and interaction analyses.

This has been revised to the following:

This multicenter study of 20,671 older adults in China analyzed the bidirectional asymmetric and threshold-response associations between cognitive and physical function using logistic or linear regression and restricted cubic spline, with subgroup and interaction analyses.

The correction will appear in the online version of the paper on the JMIR Publications website, together with the publication of this correction notice. Because this was made after submission to PubMed, PubMed Central, and other full-text repositories, the corrected article has also been resubmitted to those repositories.
