# Supplementary material for: Assessment of Bidirectional and Threshold-Response Associations Between Cognitive Function and Physical Performance: Nationwide Cross-Sectional Study
Source: JMIR Public Health Surveill. 2025 Nov 13;11:e80575. doi: 10.2196/80575 (PMC12616459; doi:10.2196/80575)

Bidirectional and Threshold-Dependent Associations Between Cognitive Function and Physical Performance: Nationwide Multicenter Study in China Cross-Sectional Study

Supplementary materials

Supplementary Tables

一、***Supplementary Table 1***

Supplementary Table 1 Multiple Linear Regression Analysis of Cognitive Function and Physical Performance

	**SPPB** β (95%CI)	**Balance** β (95%CI)	**Gait speed** β (95%CI)	**FTSST** β (95%CI)
Crude model				
**MMSE**	0.18(0.18,0.19)***	0.06(0.06,0.06)***	0.05(0.04,0.05)***	0.08(0.07,0.08)***
**Cognitive impairment**				
No	Reference	Reference	Reference	Reference
Yes	-1.56(-1.67,-1.44)***	-0.38(-0.42,-0.34)***	-0.55(-0.60,-0.50)***	-0.63(-0.69,-0.57)***
Model 1				
**MMSE**	0.18(0.17,0.19)***	0.05(0.05,0.06)***	0.05(0.04,0.05)***	0.08(0.07,0.08)***
**Cognitive impairment**				
No	Reference	Reference	Reference	Reference

Yes	-1.38(-1.49,-1.27)***	-0.3(-.33,-0.26)***	-0.51(-0.57,-0.46)***	-0.57(-0.63,-0.51)***
Model 2				
**MMSE**	0.18(0.17,0.19)***	0.05(0.05,0.06)***	0.04(0.04,0.05)***	0.08(0.07,0.08)***
**Cognitive impairment**				
No	Reference	Reference	Reference	Reference
Yes	-1.35(-1.46,-1.24)***	-0.29(-0.33,-0.25)***	-0.5(-0.55,-0.45)***	-0.56(-0.62,-0.50)***

Note

*: p<0.05; **: p<0.01; ***: p<0.001

Crude model: unadjusted covariates

Model 1: adjusted for sociodemographic characteristics adjusted for sociodemographic characteristics (age, gender, BMI, education level, marital status, number of children, residential category, living arrangement, geographic region, data source, monthly household income).

Model 2: adjusted for Model 1 variables plus health and behavioral factors(chronic diseases, types of long-term medication, alcohol consumption, smoking, frequency of weekly social activities, frequency of weekly exercise, self-reported social support, and self-reported vision and hearing impairments).

二、*Supplementary Table 2*

Supplementary Table 2 Linear Regression Analysis of Physical Performance on Cognitive Function

	MMSE β(95%CI)	MMSE β(95%CI)

Crude model		***Crude model***	
**SPPB**	0.46(0.43,0.48)***	**SPPB group**	-1.66(-1.79,-1.53)***
**Balance**	1.3(1.24,1.36)***	**Balance group**	-2.68(-2.82,-.54)***
**Gait speed**	0.53(0.48,0.58)***	**Gait speed group**	-1.22(-1.36,-1.09)***
**FTSST**	0.68 （0.64,0.72)***	**FTSST group**	-2.13(-2.25,-2.00)***
Model 1		***Model 1***	
**SPPB**	0.39(0.37,0.41)***	**SPPB group**	-1.16(-1.28,-1.04)***
**Balance**	1.022(0.97,1.08)***	**Balance group**	-2.03(-2.16,-1.90)***
**Gait speed**	0.43 （0.39,0.48)***	**Gait speed group**	-0.89(-1.01,0.77)***
**FTSST**	0.61 （0.58,0.65)***	**FTSST group**	-1.53(-1.64,-1.41)***
Model 2		***Model 2***	
**SPPB**	0.38(0.36,0.40)***	**SPPB group**	-1.14(-1.25,-1.02)***
**Balance**	1.01(0.96,1.07)***	**Balance group**	-2.02(-2.15,-1.89)***
**Gait speed**	0.42 （0.38,0.47)***	**Gait speed group**	-0.86(-0.98,-0.74)***
**FTSST**	0.610.57,0.64)***	**FTSST group**	-1.50(-1.62,-1.39)***

Note

*: p<0.05; **: p<0.01; ***: p<0.001

Crude model: unadjusted covariates

Model 1: adjusted for sociodemographic characteristics adjusted for sociodemographic characteristics (age, gender, BMI, education level, marital status, number of children, residential category, living arrangement, geographic region, data source, monthly household income).

Model 2: adjusted for Model 1 variables plus health and behavioral factors(chronic diseases, types of long-term medication, alcohol consumption, smoking, frequency of weekly social activities, frequency of weekly exercise, self-reported social support, and self-reported vision and hearing impairments).

三、*Supplementary Table 3*

Supplementary Table 3 Association Between MMSE Inflection Points and physical performance（SPPB）

	OR(95%CI)	P-value
Crude model		
**<19**	Reference	
**19-24**	0.60(0.51,0.69)	<0.001
**≥24**	0.26(0.23,0.30)	<0.001
Model 1		
**<19**	Reference	
**19-24**	0.65(0.56,0.76)	<0.001
**≥24**	0.31(0.27,0.36)	<0.001
Model 2		
**<19**	Reference	
**19-24**	0.66(0.57,0.77)	<0.001
**≥24**	0.31(0.27,0.36)	<0.001

Note: Crude model: unadjusted covariates

Model 1: adjusted for sociodemographic characteristics adjusted for sociodemographic characteristics (age, gender, BMI, education level, marital status, number of children, residential category, living arrangement, geographic region, data source, monthly household income).

Model 2: adjusted for Model 1 variables plus health and behavioral factors(chronic diseases, types of long-term medication, alcohol consumption, smoking, frequency of weekly social activities, frequency of weekly exercise, self-reported social support, and self-reported vision and hearing impairments).

四 Supplementary Table 4

Supplementary Table 4 Interactive effect

Subgroup	Interaction β	Interaction OR(95%CI)	P	Overall interaction P-value
**Age**				**<0.001**
60-74	Reference	Reference		
75-89	0.45	1.58(1.22,2.03)	<0.001	
≥90	0.71	2.03(1.23,3.35)	0.006	
**Gender**				**0.05**
Males	Reference	Reference		
Females	-0.21	0.81(0.67,0.99)	0.038	
**BMI(kg/m²)**				**0.307**
<18.5	Reference	Reference		
18.5-24	-0.17	0.84 (0.53,1.33)	0.463	
≥24	-0.01	0.99 (0.61,1.58)	0.957	
**Education**				**<0.001**
Primary school or below	Reference	Reference		
Middle school	0.55	1.73 (1.36,2.21)	<0.001	
High school or vocational school	0.43	1.53 (1.06,2.21)	0.022	
College or above	0.77	2.17 (1.02,5.75)	0.011	
**Marital Status**				**0.174**
Other	Reference	Reference		
Married	-0.19	0.83 (0.61,1.12)	0.217	
**Children**				**0.052**
None	Reference	Reference		

One	-0.44	0.65 (0.31,1.33)	0.237	
Two	-0.30	0.74 (0.36,1.51)	0.405	
Three and more	-0.10	0.90 (0.45,1.83)	0.776	
**Residence**				**<0.001**
Rural	Reference	Reference		
Urban	0.64	1.90 (1.55,2.32)	<0.001	
**Living Arrangement**				**0.775**
Living alone	Reference	Reference		
Living with family	0.11	1.11 (0.69,1.81)	0.7	
Living with spouse	0.04	1.04 (0.65,1.66)	0.874	
**Region**				**<0.001**
East	Reference	Reference		
West	-0.36	0.70 (0.57,0.86)	<0.001	
**Data Source**				**0.625**
Home	Reference	Reference		
Community health service center	-0.050	0.95 (0.78,1.16)	0.62	
**Income(RMB/Month)**				**<0.001**
<5000	Reference	Reference		
≥5000	0.85	2.34(1.72,3.16)	<0.001	

Supplementary Figures

一、*Supplementary Figure 1，Supplementary Figure 1a、Supplementary Figure 1b*

Supplementary Figure 1
Restricted cubic spline regression analysis of MMSE with balance group

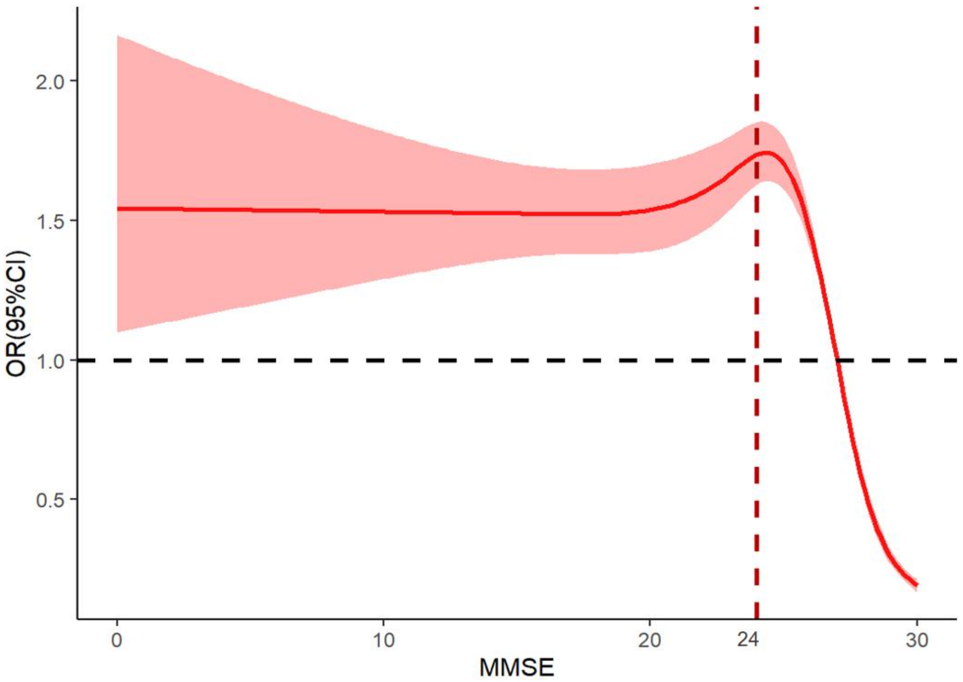

Supplementary Figure 1a Gender stratification

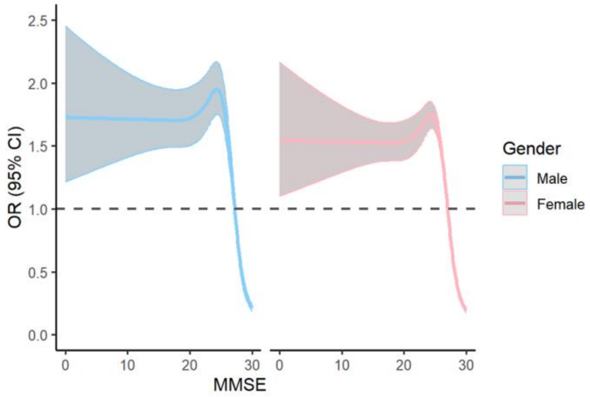

Supplementary Figure 1b Education level stratification

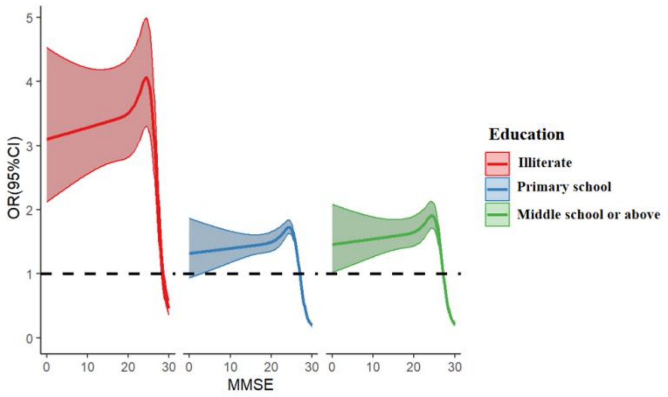

二、*Supplementary Figure 2、Supplementary Figure 2a、Supplementary Figure 2b*

Supplementary Figure 2
Restricted cubic spline regression analysis of MMSE with gait group

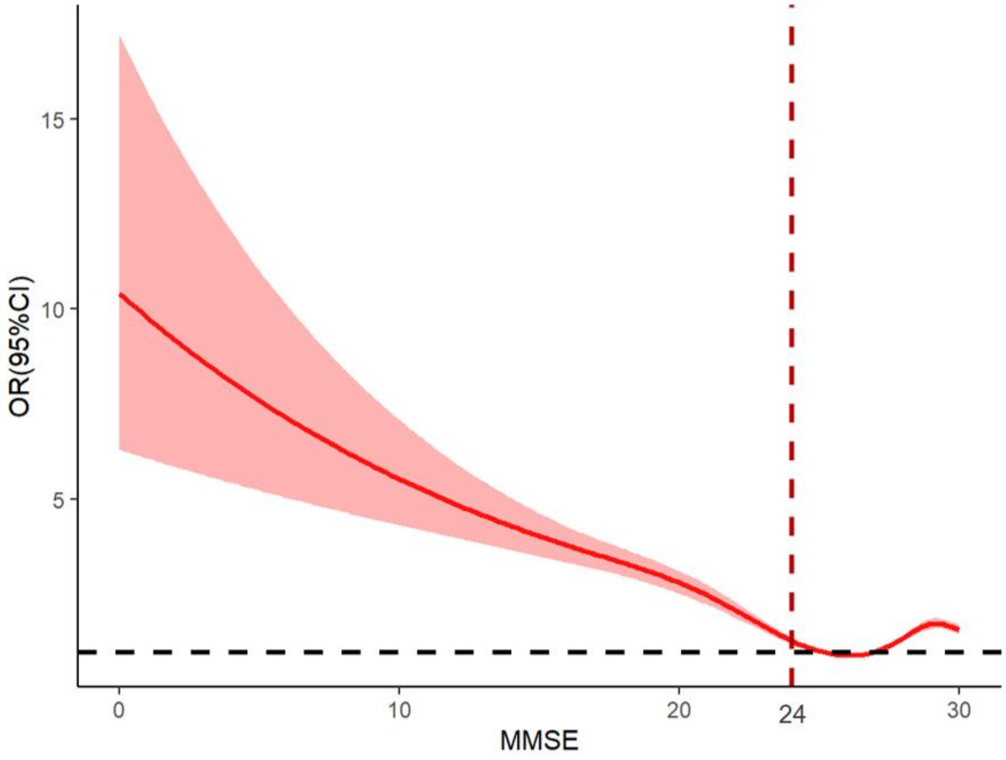

Supplementary Figure 2a Gender stratification

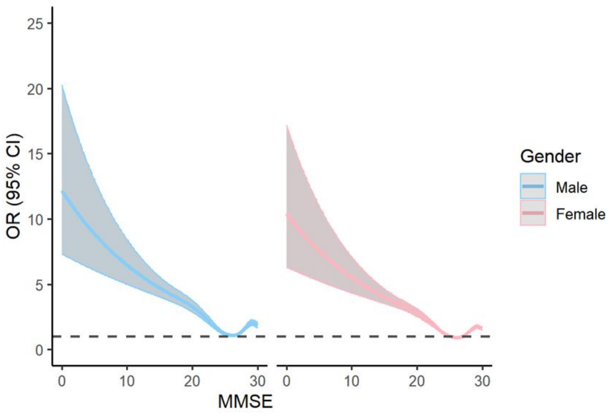

Supplementary Figure 2b Education level stratification

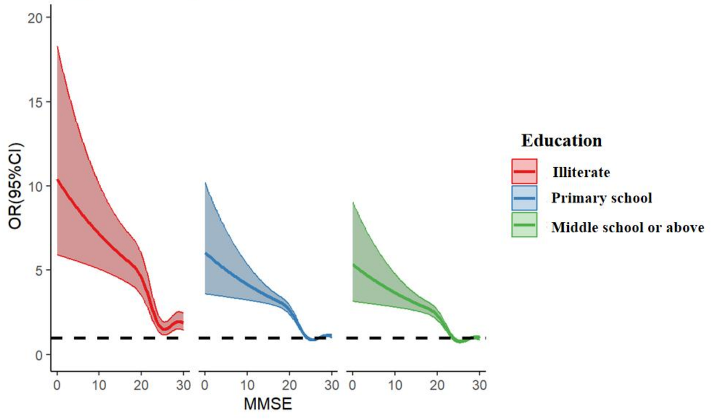

三、***Supplementary Figure 3***、***Supplementary Figure 3a***、***Supplementary Figure 3b***

Supplementary Figure 3
Restricted cubic spline regression analysis of MMSE with FTSST group

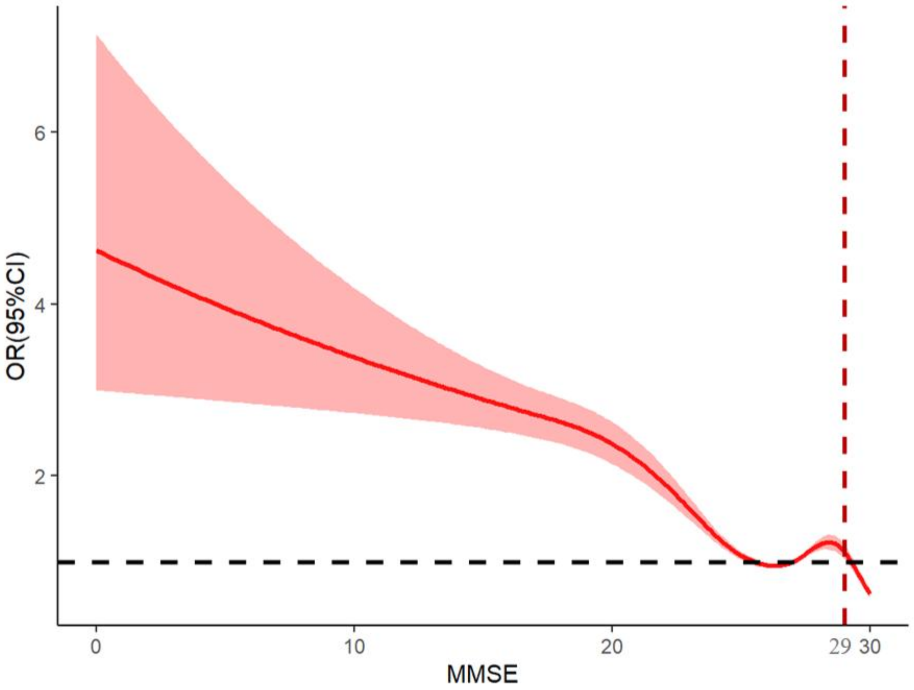

Supplementary Figure 3a Gender stratification

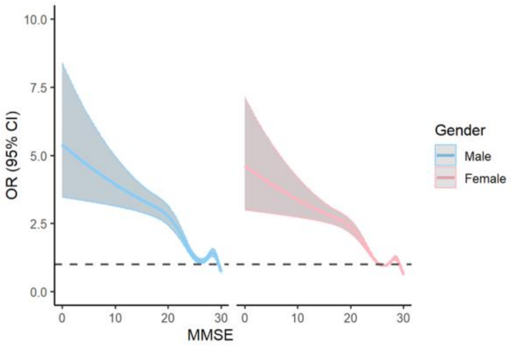

Supplementary Figure 3b Education level stratification

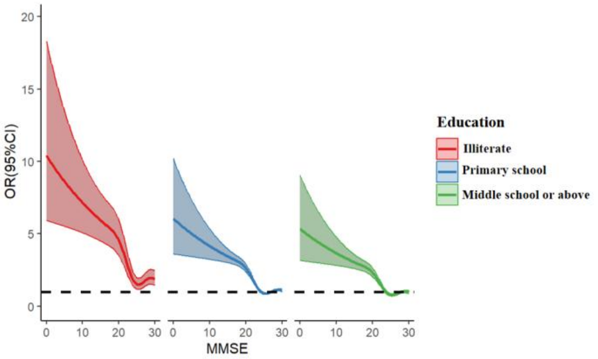

四、*Supplementary Figure 4*

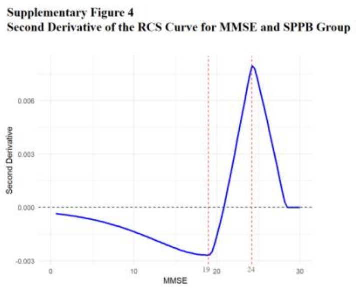

Supplement: Multimedia Appendix 1 [file publichealth-v11-e80575-s001.pdf]
